# A 4-miRNA signature to predict survival in glioblastomas

**DOI:** 10.1371/journal.pone.0188090

**Published:** 2017-11-14

**Authors:** Simon K. Hermansen, Mia D. Sørensen, Anker Hansen, Steen Knudsen, Alvaro G. Alvarado, Justin D. Lathia, Bjarne W. Kristensen

**Affiliations:** 1 Department of Pathology, Odense University Hospital, Odense, Denmark; 2 Department of Clinical Research, University of Southern Denmark, Odense, Denmark; 3 Medical Prognosis Institute, Hørsholm, Denmark; 4 Department of Cellular and Molecular Medicine, Lerner Research Institute, Cleveland Clinic, Cleveland, Ohio, United States of America; 5 Department of Molecular Medicine, Cleveland Clinic Lerner College of Medicine of Case Western Reserve University, Cleveland, Ohio, United States of America; University of South Alabama Mitchell Cancer Institute, UNITED STATES

## Abstract

Glioblastomas are among the most lethal cancers; however, recent advances in survival have increased the need for better prognostic markers. microRNAs (miRNAs) hold great prognostic potential being deregulated in glioblastomas and highly stable in stored tissue specimens. Moreover, miRNAs control multiple genes representing an additional level of gene regulation possibly more prognostically powerful than a single gene. The aim of the study was to identify a novel miRNA signature with the ability to separate patients into prognostic subgroups. Samples from 40 glioblastoma patients were included retrospectively; patients were comparable on all clinical aspects except overall survival enabling patients to be categorized as short-term or long-term survivors based on median survival. A miRNome screening was employed, and a prognostic profile was developed using leave-one-out cross-validation. We found that expression patterns of miRNAs; particularly the four miRNAs: hsa-miR-107_st, hsa-miR-548x_st, hsa-miR-3125_st and hsa-miR-331-3p_st could determine short- and long-term survival with a predicted accuracy of 78%. Heatmap dendrograms dichotomized glioblastomas into prognostic subgroups with a significant association to survival in univariate (HR 8.50; 95% CI 3.06–23.62; p<0.001) and multivariate analysis (HR 9.84; 95% CI 2.93–33.06; p<0.001). Similar tendency was seen in The Cancer Genome Atlas (TCGA) using a 2-miRNA signature of miR-107 and miR-331 (miR sum score), which were the only miRNAs available in TCGA. In TCGA, patients with O6-methylguanine-DNA-methyltransferase (MGMT) unmethylated tumors and low miR sum score had the shortest survival. Adjusting for age and MGMT status, low miR sum score was associated with a poorer prognosis (HR 0.66; 95% CI 0.45–0.97; p = 0.033). A Kyoto Encyclopedia of Genes and Genomes analysis predicted the identified miRNAs to regulate genes involved in cell cycle regulation and survival. In conclusion, the biology of miRNAs is complex, but the identified 4-miRNA expression pattern could comprise promising biomarkers in glioblastoma stratifying patients into short- and long-term survivors.

## Introduction

Glioblastomas are the most common primary malignant brain tumors in adults. Patients diagnosed with glioblastoma have a poor prognosis, but improvements in overall survival have been made over the last decade [1, 2] increasing the necessity for better prognostic markers. Histology combined with new molecular techniques is now the gold standard in glioma diagnostics [3]; as several molecular alterations have proved to be important as diagnostic and prognostic tools e.g. mutations in the isocitrate dehydrogenase 1/2 (IDH1/2) genes and the promoter of telomerase reverse transcriptase (TERT) as well as methylations of the O6-methylguanine-DNA-methyltransferase (MGMT) promoter [3, 4]. However, glioblastoma patients with tumors of similar histological appearance and molecular pattern still show great differences in overall survival. Better separation of patients could help select candidates for more aggressive treatment and active rehabilitation.

A group of non-coding RNAs called microRNAs (miRNAs) can base-pair to target messenger RNA (mRNA) causing translational repression or mRNA degradation based on the level of complementarity between strands. miRNAs originate from endogenous miRNA gene transcripts (pri-miRNAs) or from introns of protein-coding genes [5]. In mammalian cells, miRNAs mainly inhibit mRNA translation under imperfect binding to miRNA-recognition elements (MRE) within the 3’-untranslated region (UTR) of target mRNAs [6, 7].

miRNAs are excellent biomarker candidates as they are more robust than mRNA [8–11], are deregulated in glioblastomas [12], and may control numerous targets [13]. Furthermore, global expression profiling of miRNAs generates more simple data sets than mRNA (2000 miRNAs vs. >40000 mRNAs). Several miRNAs and miRNA signatures have been interrogated to evaluate their diagnostic, prognostic, predictive and/or therapeutic potential in glioblastomas as recently reviewed by Areeb et al. [14]. Dependent on their prognostic impact, some miRNAs have been characterized as pro-oncogenic and others as tumor-suppressive. High levels of miR-21 [15–17], miR-182 [18], and miR-196a/miR-196b [19] as well as low levels of miR-181b [16], miR-195 [20], and miR-196b [20] have been associated with poor prognosis in glioma. miR-196a/b has been found to hold both positive and negative prognostic impact [19, 20]. miRNA signatures, comprised of a combination of miRNAs, have been suggested for prediction of patient prognosis, but signatures found by different studies share no or only few common miRNAs [21–25] warranting further investigation to enable clinical usefulness.

To find new biomarkers allowing separation of prognostic subgroups in glioblastoma, we profiled 2016 miRNAs in 40 patients using formaldehyde-fixed and paraffin-embedded (FFPE) material. Biomarkers developed using FFPE samples have several advantages being available in large amounts and readily accessible for retrospective studies. Since FFPE is the most common way to process tissue in routine pathology, these biomarkers can subsequently be applied to most patient samples. We utilized the leave-one-out cross-validation (LOOCV) method in training and validation sets as well as the entire cohort to produce the best prognostic profile and for most efficient use of data [26].

## Materials and methods

### Patients

Investigation was carried out using FFPE sections from 40 glioblastoma patients who underwent initial surgical resection between December 1992 and April 2005 at the Department of Neurosurgery, Odense University Hospital, Denmark. No treatment was received prior to surgery. Eligible patients had a documented survival of at least 5 months from initial diagnosis to reduce the impact of post-surgical complications. Patients were categorized as short-term (STS) or long-term survivors (LTS) based on the median survival (13 months), and difference in survival between the two groups was significant using Student’s t-test (*P* < 0.0001). The pathological specimens had ≥60% vital tumor tissue and a minimum tumor tissue area of 20 mm^2^. Two neuropathologists diagnosed all samples according to the World Health Organization 2007 guidelines [27].

The use of human tissue was approved by the official Danish ethical review board named the Regional Scientific Ethical Committee of the Region of Southern Demark (Project-ID: S2DO9Oo8O) and the official Danish data registration authority named the Data Protection Authority (file number: 2009-41-3070) and was performed in accordance with the Declaration of Helsinki. As this study was retrospective using archival brain tumor tissue, no written or verbal consent should be obtained, and none of the patients had prohibited the use of their tissue according to the Danish Tissue Application Register.

### Immunohistochemistry

mIDH1 status was determined using the BenchMark Ultra instrument (Ventana Medical Systems, Inc) with anti-IDH1 R132H H09 antibody (1:100, Dianova) as previously described [28].

### Tissue preparation

Fresh tissue biopsies and cell cultures were fixed in 4% neutral buffered formaldehyde and subsequently paraffin embedded. Four 20 μm sections were cut from each specimen, placed in RNase-free cryotubes, and stored at -20°C.

### RNA extraction and purification

Total RNA was extracted from FFPE sections using the RecoverAll™Total Nucleic Acid Isolation Kit (Ambion, AM1975) according to manufacturer’s protocol.

### Microarray

RNA was biotin-labeled using the FlashTag™ Biotin HSR RNA Labeling Kit (Affymetrix). An input of 400 nanograms total RNA was used for each reaction. Hybridization, washing and staining were performed using the Affymetrix GeneChip Hybridization, Wash and Stain Kit. All samples were hybridized to the Affymetrix GeneChip miRNA 2.0 Array, which returns expression data of 1105 human mature miRNAs and 911 precursor miRNAs (miRBase v. 15). We have used the Affymetrix platform in several published studies [29–33]. Expression data was normalized using the robust multi-array average (justRMA) method where the raw intensity values are background-corrected, log2-transformed and then quartile-normalized [34]. A linear model was fit to the normalized data to obtain an expression measure for each probe set on each array.

### Experimental and statistical setup

**Fig 1:**
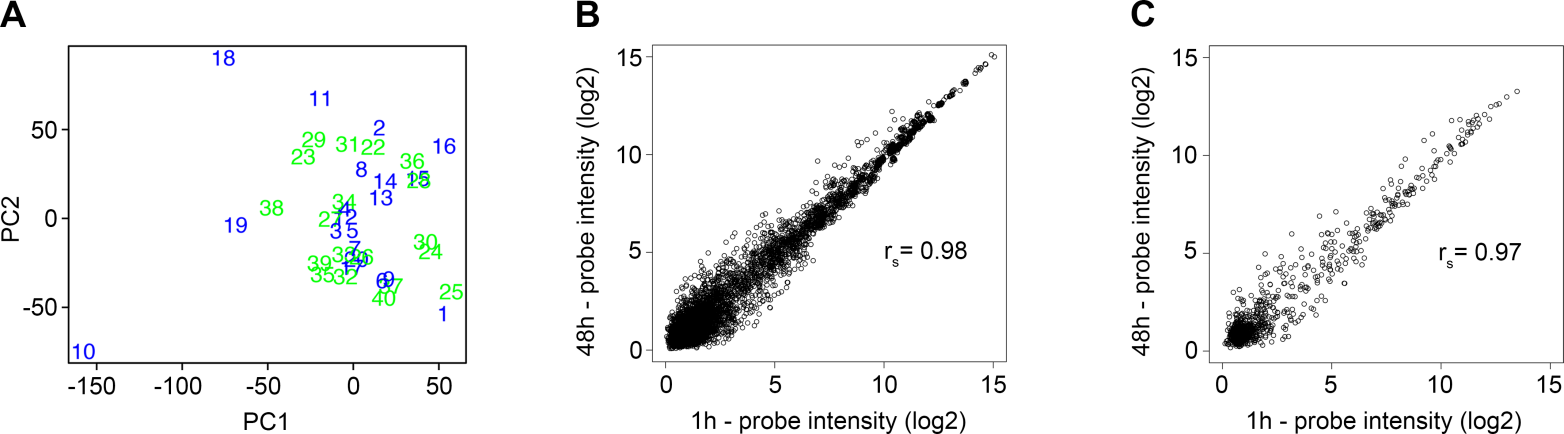
Principal component analysis and effect of fixation time. **(A)** Principal component analysis (PCA) plot showing differences between patients. The data supports our initial observation that sample 10 was a technical outlier. **(B, C)** Fixation time did not significantly affect the miRNA array data as miRNA data, obtained from a glioblastoma short-term fixated 1 hour and 48 hours, showed a strong correlation for (B) all probes (r_s_ = 0.98) and (C) human probes (r_s_ = 0.97).

Useable miRNA expression data were obtained from 39 patients. One sample (sample 10) was omitted due to low intensity on chip and categorization as an outlier in the principal component analysis (**Fig 1A**). Another sample (sample 21) had mutated IDH1 (mIDH1) and was omitted from further analysis due to potential confounding of the results [35, 36]. Patients were allocated in two sets: a training set and an independent validation set. The training set consisted of 19 patients of which ten were STS (overall survival 5–9 months) and nine LTS (overall survival > 17 months). The lower cutoff of 9 months and upper cutoff of 17 months was determined based on the median survival of glioblastoma patients being between 12 and 14 months [37], thus both cutoffs are approx. 3 months below and above the reported median survival, respectively. The validation set comprised 19 patients with continuous survival (overall survival 5–21 months). The purpose of the training set was to identify which miRNAs were associated with survival followed by testing the prognostic value of the miRNAs on the validation set. The prognostic miRNA profile was generated from the training set using LOOCV utilizing Student’s t-test for selecting probes in each loop. The t-test compared the expression of genes in LTS and STS patients and ranked genes according to their t-statistic. The number of genes to use for prediction was determined in a nested LOOCV using the inner loop to determine the optimal number of genes and the outer loop to test the performance of the optimally selected genes by a support vector machine (r-project.org package e1071). The nesting was necessary to avoid overfitting the model to the data. The resulting prognostic profile was applied as a support vector machine to the validation set of to make the validation as generalizable as possible. It categorized patients as STS or LTS. Results were subsequently checked against the clinical data. Overall survival based on the prediction was compared using log-rank testing, and prognosticator accuracy was calculated using Fisher’s exact test. An association with overall survival was tested using an unsupervised approach where the prognosticator sorted the validation set without prior grouping into STS and LTS. A heatmap of top ten deregulated miRNAs was generated using Euclidean distance measure and hierarchical clustering. Overall survival between patterns was compared using log-rank testing and Cox proportional hazard regression analysis. A volcano plot was generated to visualize important changes between STS and LTS datasets. Subsequently, LOOCV was carried out using all eligible samples, and the accuracy of the predictive model was calculated. STS and LTS groups were balanced with regards to age, treatment, extent of resection, and age of FFPE material (**Table 1**). Difference between means was computed using one-way analysis of variance (ANOVA). The raw microarray data files and data underlying the survival analysis have been deposited at Gene Expression Omnibus (GEO) under accession number GSE104554.

**Table 1:** Glioblastoma patient characteristics.

Parameter	Training set		Validation set
	STS (n = 10)	LTS (n = 9)	Continuous (n = 19)
**Patient age**			
Mean (range)	59.6 (49.0–68.0)	56.8 (37.0–72.0)	55.8 (42.0–69.0)
**Overall survival (months)**			
Mean (range)	7 (5–9)	21 (17–32)	13 (5–21)
**Radiation**			
Yes	8	8	19
No	1	1	0
Unknown	1	0	0
**Temozolomide**			
Concomitant	0	0	0
Adjuvant (at tumor relapse)	0	4	1
Unknown	0	0	0
**Resection**			
Partial	4	5	11
Radical	4	3	5
Unknown	2	1	3
**Tumor tissue (%)**			
Mean (range)	77.0 (60.0–100.0)	82.8 (70.0–100.0)	78.4 (60.0–100.0)
**Specimen age**			
Mean (range)	13.5 (8.4–19.7)	12.4 (7.5–17.6)	13.1 (7.1–20.5)

*Abbreviations*: LTS: long-term survivors; STS short-time survivors

### Cell cultures and fixation experiment

A glioblastoma short-term culture, T78, established in our laboratory [38], was cultured in serum-free medium and grown as spheroids as previously described [39]. To test the influence of fixation time, we did a correlation test between miRNA profiles of the glioblastoma short-term culture that underwent fixation for 1 hour, 12, 24, or 48 hours. An acceptable Spearman correlation existed between miRNA arrays at 1 hour and 48 hours (r_s_ = 0.98 all probes, r_s_ = 0.97 human probes) validating that fixation time did not affect the results (**Fig 1B and 1C**).

### The Cancer Genome Atlas (TCGA)

miRNA signatures were derived for 533 patients in The Cancer Genome Atlas (TCGA [40], available at https://tcga-data.nci.nih.gov/tcga/) based on expression of miR-107 and miR-331. Clinical data was retrieved from the study by Brennan et al [41]. To make the TCGA dataset comparable to our dataset, only patients with primary glioblastoma, wildtype IDH1 (wtIDH1), and an overall survival between 5–33 months were included in the analysis (n = 247). Patient characteristics of the complete set used in this study (n = 38) and the TCGA dataset (n = 247) is presented in **S1 Table**. Of the 247 patients, MGMT status was available for only 180 patients. Using the median as cutoff value, patients were divided into those with a high or low score based on the expression levels of miRNA-107, miRNA-331, and the summed expression levels of miRNA-107 and miRNA-331s (miR sum score). Differences in survival were analyzed with log-rank testing. Multivariate analysis was performed including the following variables: age at time of surgery, MGMT status, and miR sum score. Difference in miR sum score between patients with methylated and unmethylated tumors was compared using Student’s unpaired t-test. Data underlying the statistical analysis is available in **S1 File**.

### Target prediction and pathway analysis

Targets of the four hsa-miRNAs used in the 4-miRNA signature were predicted with DIANA-miRPath v.3 provided by the DIANA-microT-CDS algorithm and the Kyoto Encyclopedia of Genes and Genomes (KEGG) pathway [42–44].

### Statistics

Statistical analyses were performed in Prism 5.0 (GraphPad Software, Inc.), STATA (StataCorp LP), and R (affy package from Bioconductor [45]). *P*-values <0.05 were considered significant.

## Results

### Developing the prognostic profile

We profiled miRNA expression levels in 40 glioblastoma patients of which 38 qualified for subsequent analyses (**Tables 1 and 2**). The prognostic profile was developed on the 19 eligible samples in the training set using LOOCV, where a single patient is used as validation and the remaining patients as the training set. This was repeated until every patient had been used once for validation generating a list of optimal predictor miRNAs for each sample. Lastly, selecting the miRNAs present in all lists resulted in an aggregated and final list of miRNAs. The LOOCV yielded a prognostic gene list consisting of ten miRNAs (hsa-miR-107_st, hsa-miR-3125_st, hsa-miR-331-3p_st, hp_hsa-mir-4315-2_s_st, hsa-miR-548x_st, hsa-miR-3126-5p_st, hp_hsa-mir-885_st, hsa-miR-4270_st, hsa-miR-103_st, hsa-miR-887_st) with a predicted accuracy of 68%. However, when tested against the validation set, this prognosticator predicted only one LTS correctly. The rest was predicted as STS (log-rank: *P* = 0.75; Fischer’s exact test: *P* = 0.47) corresponding to an accuracy of 58% (11 correct, 8 wrong). Due to the relatively small training and validation sets and very heterogeneous tumors, the accuracy of the predictive model was investigated using all 38 eligible samples. Applying the LOOCV approach to all 38 samples, the model predicted an accuracy of 78% with hsa-miR-107_st, hsa-miR-548x_st, hsa-miR-3125_st and hsa-miR-331-3p_st as optimal predictors. The wrongly categorized patients were STS categorized as LTS corresponding to false negatives (type 2 errors). The four miRNAs were all downregulated in STS (**Table 2**), and low levels of each of the four miRNAs were significantly associated with poorer prognosis both using the median as a predefined cutoff (**S1 Fig**) and the optimal cut-point (**S2 Fig**).

**Table 2:** Glioblastoma patient characteristics.

ProbeID	Fold change	*P*-value (unadjusted)
**hsa-miR-107_st**	**-0.3050**	**0.000024**
**hsa-miR-3125_st**	**-0.3397**	**0.000092**
**hsa-miR-331-3p_st**	**-1.0500**	**0.000101**
hp_hsa-mir-4315-2_s_st	-0.2556	0.000172
**hsa-miR-548x_st**	**-1.2050**	**0.000204**
hsa-miR-3126-5p_st	-0.6107	0.000227
hp_hsa-mir-885_st	0.3883	0.000292
hsa-miR-4270_st	1.1590	0.000676
hsa-miR-103_st	-0.3822	0.000705
hsa-miR-887_st	-0.7126	0.000873

The ten most significantly deregulated miRNAs in the 38 glioblastomas comparing STS to LTS depicted as fold change relative to LTS. Using the leave-one-out cross validation approach, the model predicted an accuracy of 78% with hsa-miR-107_st, hsa-miR-548x_st, hsa-miR-3125_st and hsa-miR-331-3p_st as optimal predictors (indicated with **bold**).

### STS and LTS have different miRNA profiles

**Fig 2:**
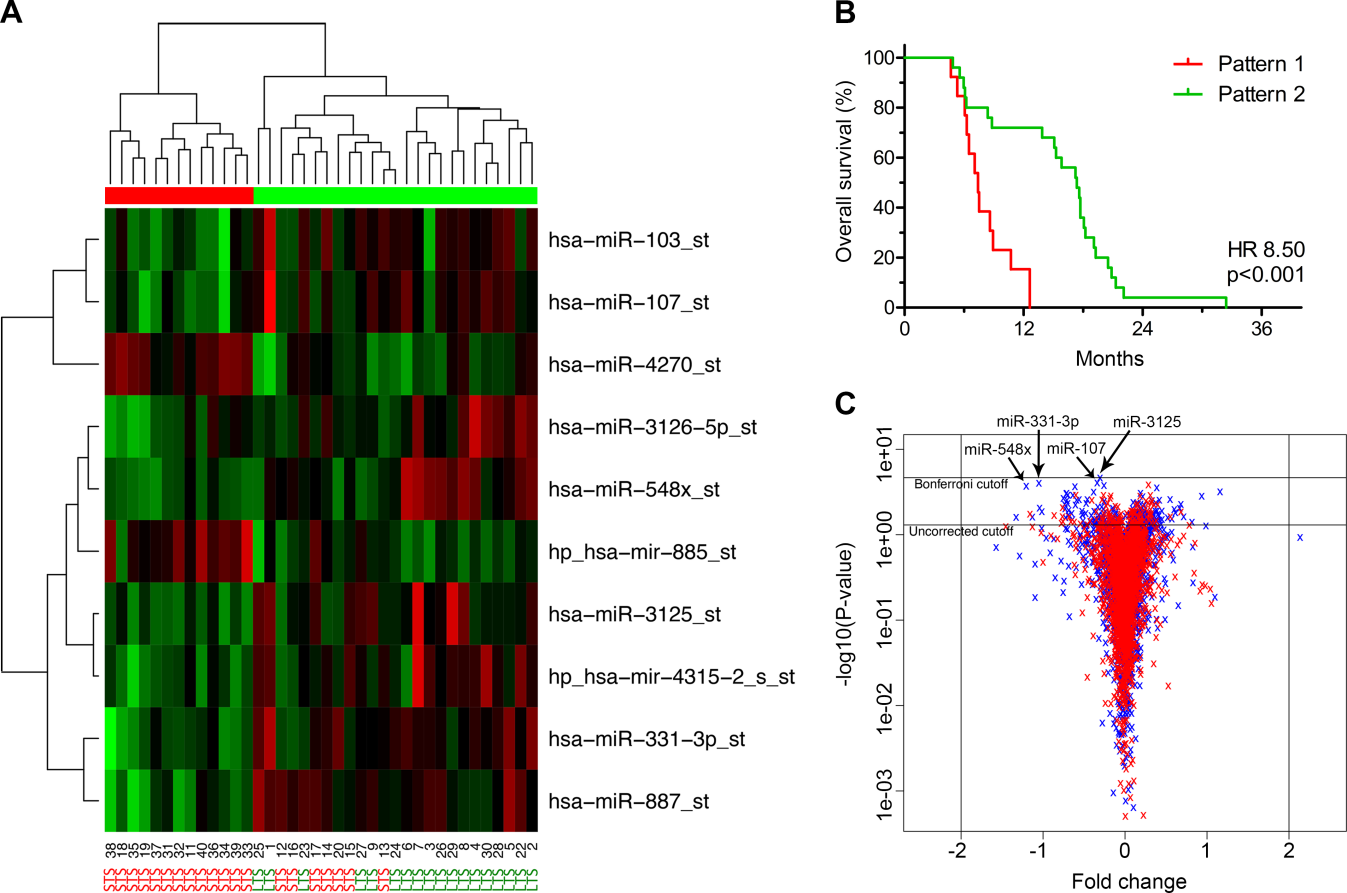
Short-(STS) and long-term (LTS) glioblastoma survivors have different microRNA (miRNA) profiles. **(A)** Heatmap of the ten most deregulated miRNAs in STS and LTS. STS and LTS are grouped into two overall patterns as shown by the dendrograms. Pattern one (red bar) was characterized by STS whereas pattern two (green bar) mostly characterized LTS (18 LTS and 7 STS). In the heatmap, red represents upregulated miRNAs and green represents downregulated miRNAs. **(B)** Kaplan Meier plot showing a significant separation in overall survival between the two patterns. **(C)** Volcano plot illustrating that no miRNAs were significantly deregulated above the two-fold threshold. Blue represent normal fold changes and p-values while red represent permutated values. The four miRNAs included in the signature are indicated with arrows.

Plotting the ten most deregulated miRNAs in a heatmap with dendrograms suggested that two overall patterns existed within the glioblastomas (**Table 2 and Fig 2A**). Pattern one was characterized by 13 patients with an overall survival shorter than 13 months, i.e. STS, whereas pattern two mostly characterized patients with an overall survival longer than 13 months, i.e. LTS, and included 18 LTS and 7 STS. The Kaplan Meier plot and log-rank statistics showed a significant separation in overall survival between the two patterns (Hazard ratio (HR) 8.50; 95% confidence interval (CI) 3.06–23.62; *P* < 0.001) (**Fig 2B**), also independent of age, second line treatment, and degree of resection (HR 9.84; 95% CI 2.93–33.06; *P* < 0.001) (**Table 3**). The two patterns showed greater prognostic impact compared to the single miRNAs in the 4-miRNA signature (**S1 and S2 Figs**). Yet, no miRNAs were above the two-fold threshold while being statistically significant in the t-test before or after Bonferroni adjustment for multiple testing (**Fig 2C**).

**Table 3:** The two miRNA patterns and multivariate analysis.

Variable		Hazard Ratio (95% CI)	*P*-value
**Pattern**	1/2	9.84 (2.93–33.06)	**<0.001**
**Age**	Continuous	0.99 (0.95–1.03)	0.59
**Radiation therapy**	No/Yes	1.51 (0.18–12.76)	0.70
**Chemotherapy**	No/Yes	1.74 (0.62–4.85)	0.29
**Resection**	Partial/Complete	0.77 (0.33–1.79)	0.54

**Fig 3:**
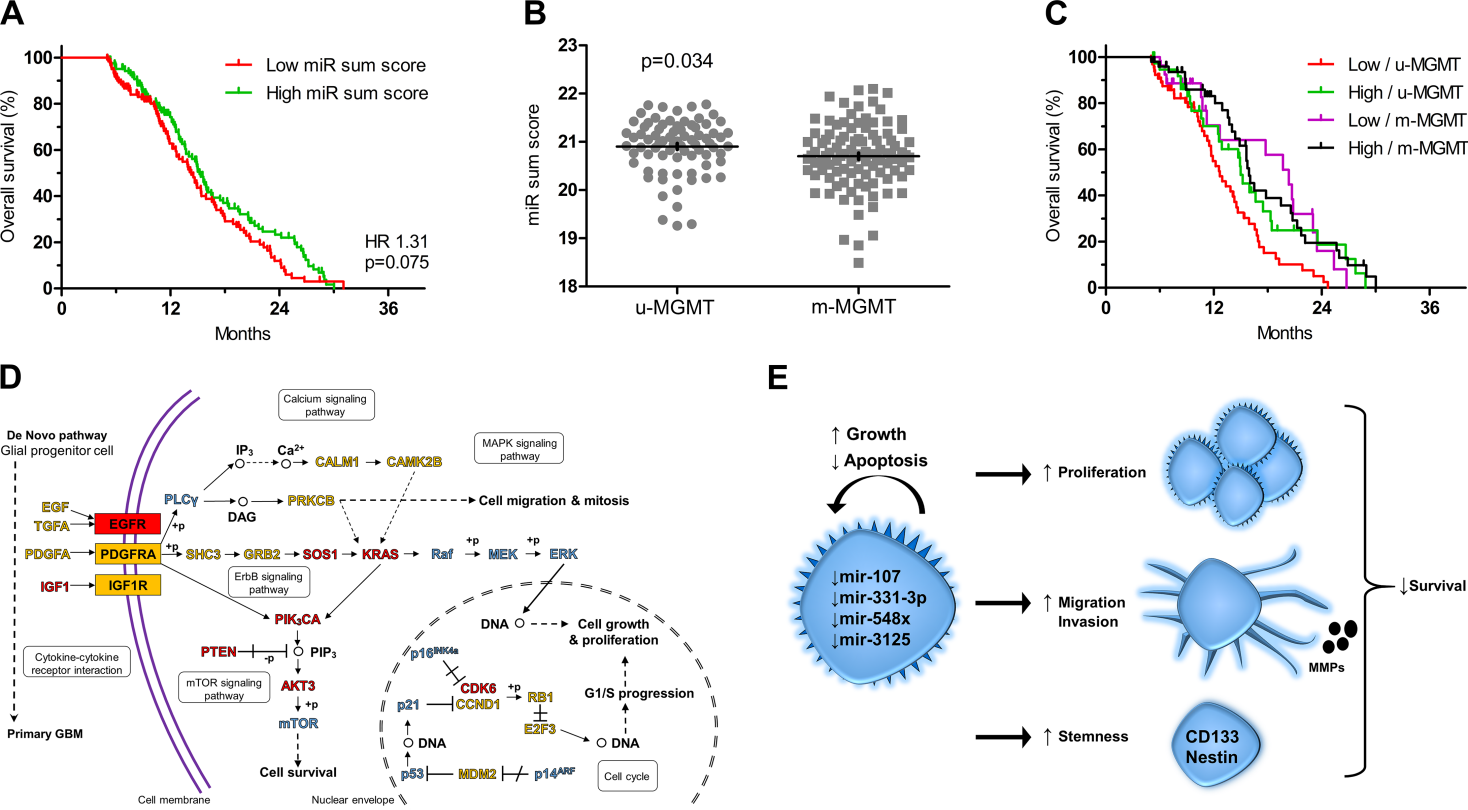
In silico gene expression analysis and KEGG pathway analysis. **(A)** Low summed scores for miR-107 and miR-331-3p (miR sum score) tended to associate with poorer prognosis in the TCGA data set (n = 247) when dichotomized at the median. **(B)** Glioblastomas with unmethylated MGMT promoter (u-MGMT) had higher miR sum score than glioblastomas with methylated MGMT promoter (m-MGMT). **(C)** Stratified into groups based on MGMT methylation status and miR sum score, patients with low miR sum score and u-MGMT had the shortest survival. **(D)** KEGG pathway analysis based on predicted targets of hsa-miR-107, hsa-331-3p, hsa-548x and hsa-3125 in the Glioma Pathway performed using the DIANA-mirPath tool. Genes regulated by at least two miRNAs are indicated with **red**, genes regulated by one miRNA with **yellow**, and genes not regulated by any of the miRNAs with **blue**. **(E)** Possible mechanisms by which downregulation of the 4-miRNA signature contribute to shorter survival in patients with glioblastoma.

To further investigate the role of the miRNA profile, we evaluated the TCGA database with data available for miR-107 and miR-331. The included patients were all diagnosed with primary glioblastoma, had wtIDH1 and an overall survival between 5–33 months. No significant difference in overall survival was found for miR-107 or miR-331 alone (**S3 Fig**). However, combining the two miRNAs into a summed score, patients with low miR sum score tended to have a poorer survival than patients with high sum score (HR 1.31; 95% CI 0.97–1.77; *P* = 0.075) (**Fig 3A**). When only including patients with known MGMT status in the analysis, low miR sum score was significantly associated with poorer outcome (HR 1.59; 95% CI 1.09–2.30; *P* = 0.014), and looking at the relation between the sum score and MGMT methylation status, unmethylated tumors had significantly higher miR sum score compared to methylated tumors (*P* = 0.034) **(Fig 3B**). Patients with low sum score and unmethylated tumors had the shortest overall survival compared to patients with high sum score and unmethylated tumors (*P* = 0.025), patients with low sum score and methylated tumors (*P* = 0.014) as well as patients with high sum score and methylated tumors (*P* = 0.001) (**Fig 3C**). Adjusting for age and MGMT status, low miR sum score was independently associated with poorer prognosis (HR 1.52; 95% CI 1.04–2.22; *P* = 0.029) (**Table 4**).

**Table 4:** TCGA and multivariate analysis.

Variable		Hazard Ratio (95% CI)	*P*-value
**Age**	Continuous	1.02 (1.00–1.03)	**0.007**
**MGMT status**	u-MGMTm-MGMT	0.68 (0.46–1.00)	**0.045**
**miR sum score**	High/Low	1.52 (1.04–2.22)	**0.029**

### KEGG pathway analysis

Target genes of the 4-signature miRNAs were predicted using DIANA-miRPath [42–44]. In the Glioma Pathway, genes involved in ErbB, mTOR, and MAPK signaling pathways as well as genes important for cell cycle were found to be most likely regulated by the miRNAs e.g. Kirsten rat sarcoma viral oncogene homolog (KRAS) (miR-3125, miR-548x-3p, miR-548x-5p), phosphatase and tensin homolog (PTEN) (miR-548x-3p, miR-548x-5p), V-akt murine thymoma viral oncogene homolog 3 (AKT3) (miR-107, miR-548x-3p), and cyclin-dependent kinase 6 (CDK6) (miR-107, miR-548x-3p) (**Fig 3D**). Generally, the miRNAs had predicted targets in many pathways important to cancer progression e.g. angiogenesis, invasion, proliferation, and survival (**Table 5**).

**Table 5:** KEGG pathway enriched for mRNAs predicted to be targeted by miRNAs in the 4-miRNA signature.

KEGG pathway	*P*-value	# genes	# miRNAs	miRNAs
Adherens junction	6.28e-11	45	4	mir-107, -3125, -548x-3p, -548x-5p
Pathways in cancer	8.82e-11	159	4	mir-107, -3125, -548x-3p, -548x-5p
Proteoglycans in cancer	1.13e-08	68	5	mir-107, -331-3p, -3125, -548x-3p, -548x-5p
Signaling pathways regulating pluripotency of stem cells	2.01e-08	68	5	mir-107, -331-3p, -3125, -548x-3p, -548x-5p
ErbB signaling pathway	2.85e-07	45	4	mir-107, -3125, -548x-3p, -548x-5p
MAPK signaling pathway	0.009	92	5	mir-107, -331-3p, -3125, -548x-3p, -548x-5p
PI3K-Akt signaling pathway	0.016	113	4	mir-107, -3125, -548x-3p, -548x-5p
Glioma	4.95e-07	33	4	mir-107, -3125, -548x-3p, -548x-5p
Ras signaling pathway	7.08e-05	86	5	mir-107, -331-3p, -3125, -548x-3p, -548x-5p
p53 signaling pathway	0.023	28	4	mir-107, -3125, -548x-3p, -548x-5p
Cell cycle	0.016	48	4	mir-107, -3125, -548x-3p, -548x-5p

## Discussion

We investigated whether a good prognostic miRNA profile could be generated from the most deregulated miRNAs in the training set consisting of 19 eligible glioblastomas resulting in an optimal profile of ten miRNAs. Applying the LOOCV procedure to the entire dataset, the predicted accuracy of the prognostic profile was 78% using the four miRNAs hsa-miR-107_st, hsa-miR-548x_st, hsa-miR-3125_st and hsa-miR-331-3p_st. These miRNAs were all slightly downregulated in STS suggesting a protective role in glioblastomas. Examining the TCGA data based on expression of miR-107 and miR-331, low miR sum score tended to associate with poor prognosis in the univariate analysis suggesting that miR-548 and miR-3125 may be important prognosticators in the miRNA profile. However, when including only patients with known MGMT methylation status, low miR sum score was an independent negative prognostic factor. Unfortunately, only two of the four miRs included in our signature were available in the TCGA dataset, thereby preventing complete validation of our 4-miRNA signature. Further, the TCGA 2-miR sum score may be a less strong prognosticator compared to the 4-miRNA signature. Also, the miR sum score appeared to have higher prognostic value in patients with unmethylated MGMT promoters.

Currently, glioblastoma STS can to some extent be identified using molecular markers e.g. IDH, MGMT, and TERT [4]. Similarly, dividing patients into risk classes using the Radiation Therapy Oncology Group-Recursive Partitioning Analysis (RTOG-RPA) has proven applicable for differentiating between LTS and STS [46, 47]. The 4-miRNA signature presented in this study may have a potential in daily pathology to further stratify patients into high or low risk groups. Hsa-miR-107 belongs to the miR-103 family both sharing homologous precursor. In the present study, hsa-miR-107 and hsa-miR-103 were among the top ten most significantly deregulated miRNAs with similar fold changes. Hsa-miR-107 has been shown to be a glioma suppressor in functional studies, and its downregulation has been reported in gliomas, glioma cell lines, and glioma stem-like cells [48–50]. Overexpression of hsa-miR-107 in glioma cells suppressed their proliferation and their migratory, invasive, and angiogenetic capabilities by targeting p53 [50], Notch-2 [48, 49], CDK6 [50], matrix metalloproteinase (MMP)-12 [48], and vascular endothelial growth factor (VEGF) [51]. Additionally, the glioma stem cell-like markers CD133 and nestin were downregulated when hsa-miR-107 was overexpressed [48]. A possible mechanism for the downregulation of hsa-miR-107 could be its localization on the long arm of chromosome 10 (10q), which is lost in up to 80% of glioblastomas alongside other tumor suppressors e.g. PTEN [35, 52]. Alternatively, downregulation could be explained by epigenetic silencing of the hsa-miR-107 promoter as seen in pancreatic cancer [53]. Recently, expression levels of hsa-miR-107 were reported to be diminished in gliomas compared to normal brain and in high-grade gliomas compared to low-grade gliomas. Further, low expression was associated with shorter overall and progression-free survival looking at all glioma grades combined [54], overall indicating that low hsa-miR-107 is involved in tumor aggressiveness. miR-331-3p was reported to be downregulated in glioblastoma cell lines compared to normal brain, and overexpression of miR-331-3p inhibited proliferation, clonogenic growth, and migration in vitro by reducing mRNA levels of neuropilin-2 [55]. Further, miR-331-3p was shown to regulate EGFR in glioblastoma cells resulting in reduced AKT activity [56]. The two remaining miRNAs in the 4-miRNA signature are largely unknown in glioma biology. Altogether, studies and target prediction in DIANA-mirPath suggest various tumor suppressive roles in pathways relating to cell survival, migration, and mitosis [43]. Theoretically, downregulation of the miRNAs in glioblastomas may lead to more aggressive tumors thereby resulting in poorer prognosis (**Fig 3E**).

Our findings that miRNA deregulation may predict prognosis in glioblastomas are consistent with three recent studies [21–23]. However, these studies used other miRNAs to generate their prognostic profiles. We measured miRNA expression levels using the Affymetrix platform. Zhang et al. used the Illumina miRNA Expression BeadChip [23], Niyazi et al. the Geniom Biochip [21], and Srinivasan et al. TCGA data that contain profiling data originating from the Agilent Human 8x15K miRNA platform [22]. Only Zhang et al. performed cross-platform validation by comparing data obtained from TaqMan assays and the Agilent Human 8x15K miRNA platform (TCGA data). Even though we used an equal amount of training samples as Zhang et al. (40 and 41, respectively), we did not identify the same miRNAs. This may be due to different ethnic groups. Although Zhang et al. were successful in validating their 5-miRNA profile in a different ethnic group (the TCGA); these records only contained expression data for three out of five miRNAs. The dissimilar miRNA profiles could also be a result of concluding on heterogeneous tumors with heterogeneous treatment histories and inclusion of patients with overall survival of weeks.

In the present study, profiling was done on a comparable patient cohort exploiting FFPE material, the most common way to store tissue samples. The patient sets used were balanced with regards to age, treatment, extent of resection, and age of FFPE material. Based on histological verification, all specimens had high tumor percentages. The objective was to study the impact of miRNAs on tumor biology and aggressiveness rather than the response to treatment including temozolomide. Whereas most patients receive radiotherapy, elderly patients with wtIDH and MGMT-unmethylated tumors are suggested not to receive temozolomide [57, 58]. We therefore decided to use tissue from before temozolomide was introduced to the standard of care to make the interpretation of results less complex. However, our analyses from the TCGA database also suggested that the miRNAs investigated in this study primarily has a prognostic influence in patients whose tumors have unmethylated MGMT promoters, and these patients do not have a significant survival benefit from TMZ treatment [59]. Further, all patients had a documented survival of at least 5 months from initial diagnosis to avoid confounding from death due to post-surgical complications. We used the LOOCV which is more powerful for distinguishing predefined classes than the clustering approach adopted by Niyazi et al. [21]. Further, the clustering analysis does not provide valid statistical identification of differentially expressed miRNAs. A drawback in our study is the limited number of patients. Further, the fold changes of the identified miRNAs were small and may not be sufficiently deregulated to be applied as solid biomarkers. To examine the prognostic strength of this 4-miRNA signature, the results should be validated on a larger patient cohort including patients who have received multimodal treatment with surgery followed by radio-chemotherapy.

In summary, we have identified a novel miRNA signature based on an independent cohort in which all the patients are clinically treated in an identical manner. Our data suggest that future identification of glioblastoma STS and LTS may consist of evaluating expression patterns of miRNAs; particularly the expression of the four miRNAs hsa-miR-107_st, hsa-miR-548x_st, hsa-miR-3125_st and hsa-miR-331-3p_st. Heatmap dendrograms dichotomized glioblastomas into prognostic subgroups that were significantly different in uni- and multivariate analyses. Using the TCGA dataset we could validate the prognostic impact of miR-107 and miR-331 with low levels being independently associated with shorter survival. The miRNAs identified in the current study were all linked to larger signaling pathways that work controlling key cellular phenotypes.

Although various reports support a great future for miRNAs as biomarkers, major discrepancies exist across studies, and improved evaluation in the future will require standardization of methods and normalization.

## Supporting information

S1 Fig The single miRNAs in the 4-miRNA signature are associated with prognosis when dichotomized at the median value. **(A)** Low expression of hsa-miR-107 was significantly associated with shorter overall survival. **(B)** The similar association was found for hsa-miR-331-3p, **(C)** hsa-miR-548x, and (**D)** hsa-miR-3125.
(TIF)

S2 Fig The single miRNAs in the 4-miRNA signature are associated with prognosis using their respective optimal cutoff values. (**A)** Low expression of hsa-miR-107 was significantly associated with shorter overall survival. **(B)** The similar association was found for hsa-miR-331-3p, **(C)** hsa-miR-548x, and **(D)** hsa-miR-3125.
(TIF)

S3 Fig The single miRNAs in the 2-miRNA TCGA signature are not associated with prognosis using when dichotomized at the median value. **(A)** Expression levels of miR-107 did not impact survival. **(B)** miR-331 levels did not correlate significantly with overall survival.
(TIF)

S1 File Dataset generated from The Cancer Genome Atlas (TCGA). (XLSX)

S1 Table Patient characteristics in the complete dataset and The Cancer Genome Atlas (TCGA) dataset. (PDF)
