# Supplementary figures and images for: A 4-miRNA signature to predict survival in glioblastomas
Source: PLoS One. 2017 Nov 14;12(11):e0188090. doi: 10.1371/journal.pone.0188090 (PMC5685622; doi:10.1371/journal.pone.0188090)

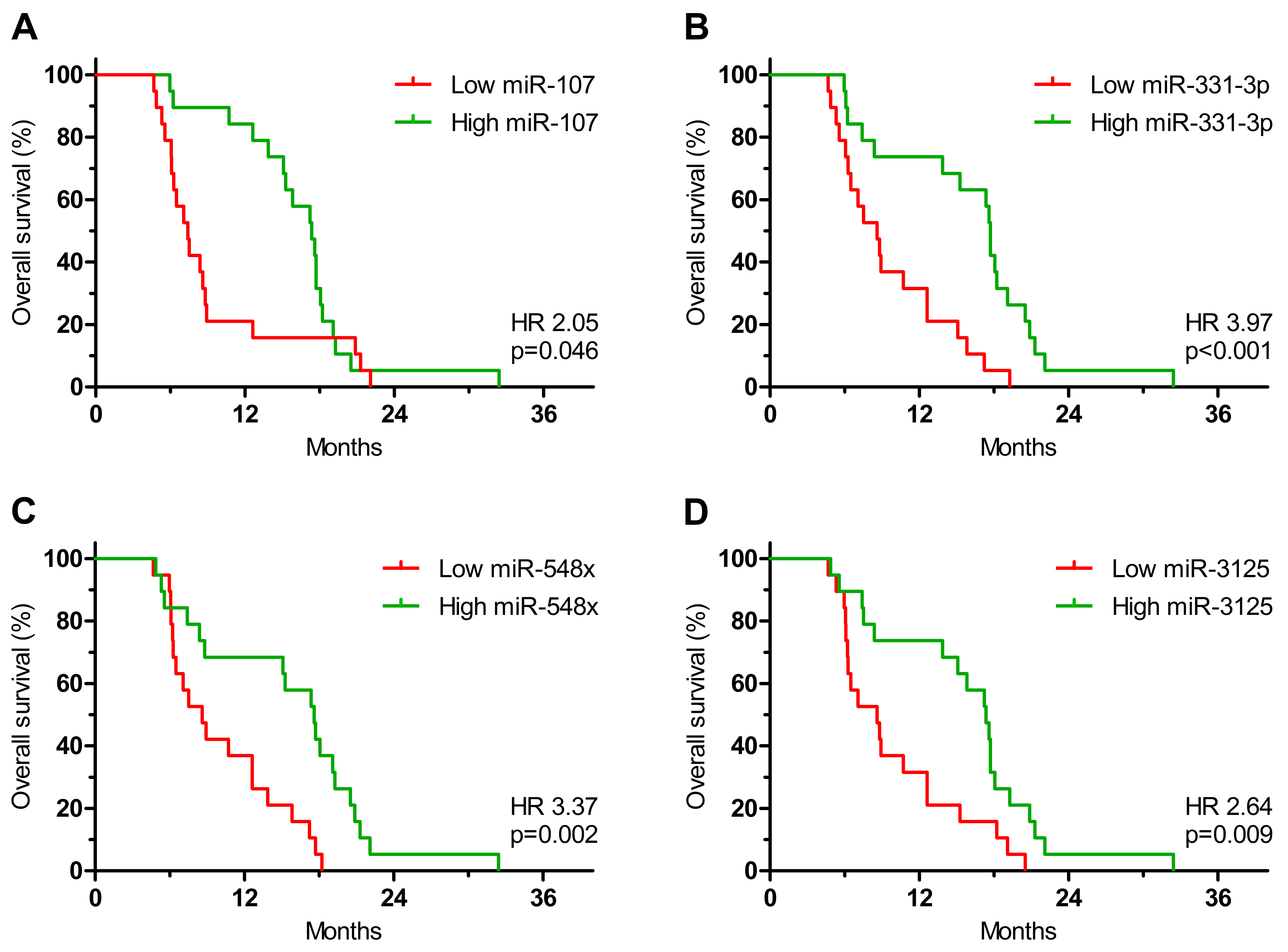

Supplement: S1 Fig — (A) Low expression of hsa-miR-107 was significantly associated with shorter overall survival. (B) The similar association was found for hsa-miR-331-3p, (C) hsa-miR-548x, and (D) hsa-miR-3125. (TIF) [file pone.0188090.s001.tif]

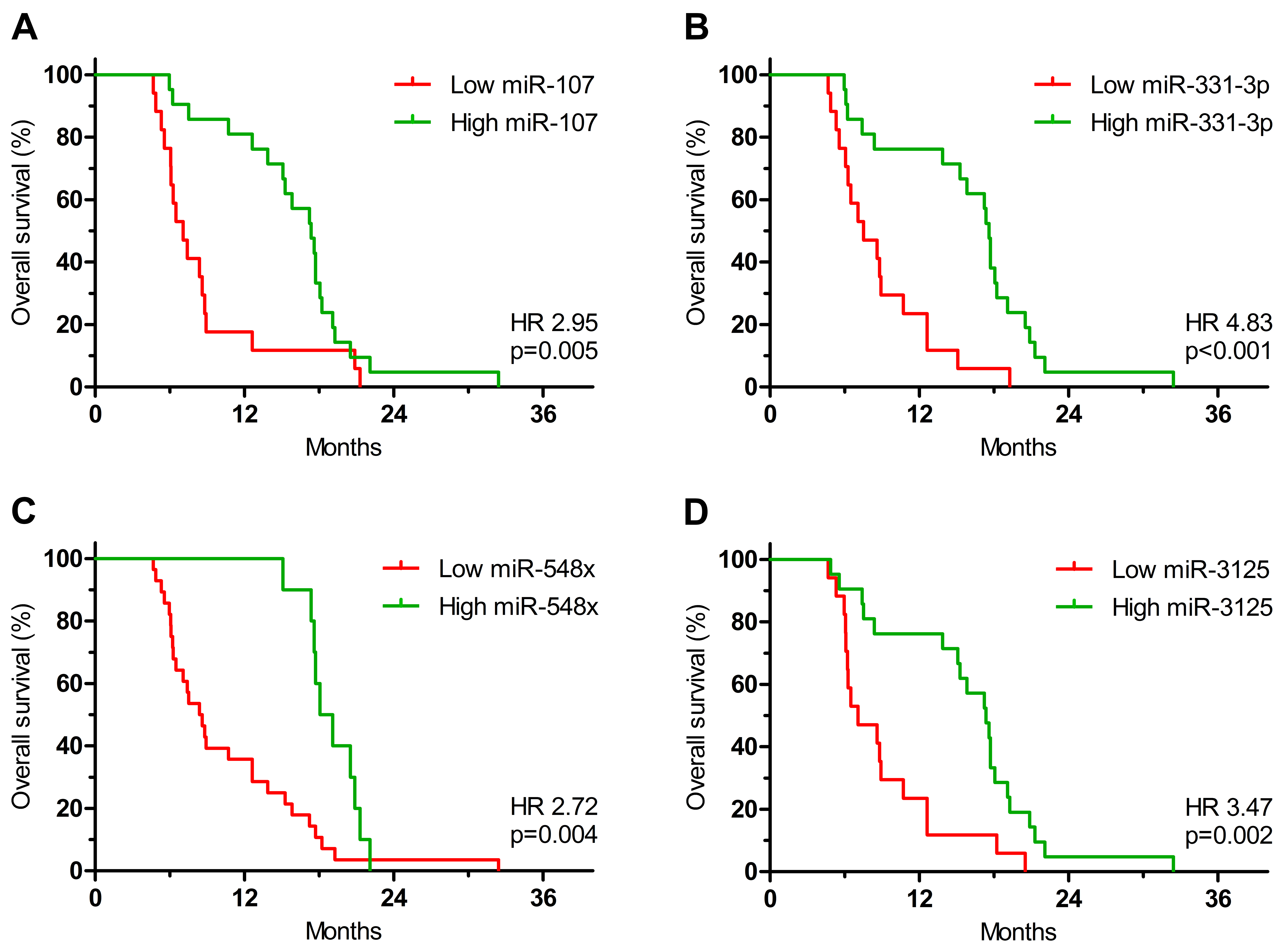

Supplement: S2 Fig — (A) Low expression of hsa-miR-107 was significantly associated with shorter overall survival. (B) The similar association was found for hsa-miR-331-3p, (C) hsa-miR-548x, and (D) hsa-miR-3125. (TIF) [file pone.0188090.s002.tif]

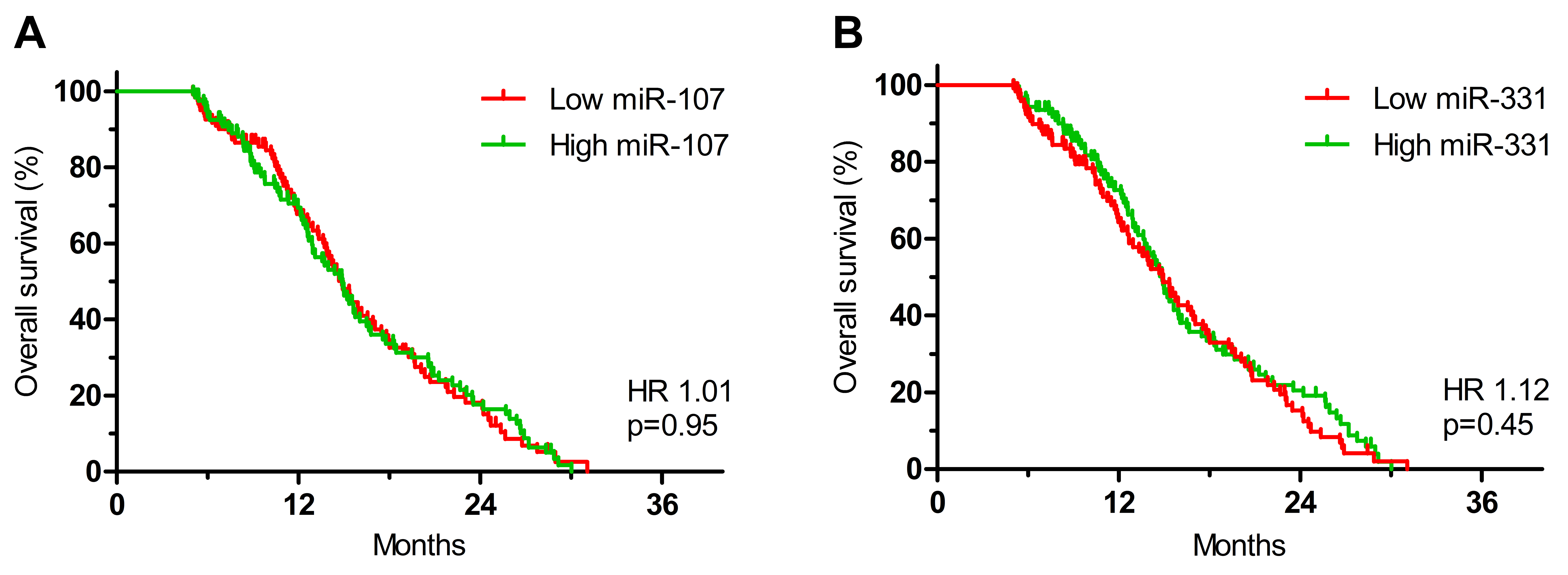

Supplement: S3 Fig — (A) Expression levels of miR-107 did not impact survival. (B) miR-331 levels did not correlate significantly with overall survival. (TIF) [file pone.0188090.s003.tif]
