# Supplementary material for: A 4-miRNA signature to predict survival in glioblastomas
Source: PLoS One. 2017 Nov 14;12(11):e0188090. doi: 10.1371/journal.pone.0188090 (PMC5685622; doi:10.1371/journal.pone.0188090)
Supplement: S1 Table — (PDF) [file pone.0188090.s005.pdf]

**S1 Table.** Patient characteristics in the complete dataset and the Cancer Genome Atlas (TCGA) dataset.

| **Parameter** | **Complete set (n=38)** | **TCGA set (n=247)** |
|---|---|---|
| **Patient age** | | |
| Mean (range) | 57.1 (37.0-72.0) | 59.8 (19-86.6) |
| **Sex** | | |
| Female (%) | 14 (37 %) | 89 (36 %) |
| Male (%) | 24 (63 %) | 158 (64 %) |
| **Overall survival (months)** | | |
| Mean (range) | 12.9 (4.7-32.4) | 13.6 (5.03-31.1) |
| **Status** | | |
| Alive | 0 | 70 |
| Dead | 38 | 177 |
| **Radiation** | | |
| Yes | 35 | 239 |
| No | 2 | 3 |
| Unknown | 1 | 7 |
| **Temozolomide** | | |
| Yes | 5* | 204 |
| No | 33 | 38 |
| Unknown | 0 | 7 |

* at tumor relapse
